# Helping non-scientists (the people who pay your salary) understand what we do

**DOI:** 10.3325/cmj.2021.62.198

**Published:** 2021-04

**Authors:** Charles H. Calisher

Most of us do not ask the plumber, carpenter, electrician, baker, auto mechanic, butcher, or other trade people how they do what they do. We do not know how to do what they do and, anyway, we do not have the time to do it (and do not want to do it). That is why we pay them to do it, particularly on a Saturday night. If the product or end result is satisfactory, we probably return to them the next time an emergency occurs. If we are not satisfied, we do not call them, we call someone else and hope for the best. We cannot do this with a physician, veterinarian, dentist or others who do things to our bodies, our family members’ bodies, or to the bodies of our domesticated or non-domesticated animals. Sometimes it is too late to have a second try at finding help.

However, there is something we can at least partially control in regard to medicine. It has been jokingly said that half of practicing physicians graduated in the lower half of their class. Not quite true but we get the point; not all clinicians are of equal expertise. So, what can we do? What do we do?

Imagine the confusion or concern of the average layperson (the taxpayer) who is seen by a clinician and told this or that and then, not knowing what the hell the physician means when s/he says s/he will remove one of the patient’s kidneys (“You have two.”) or lung, leg, ear, ovary, testicle, or eye (“You have two.”), or tonsils, appendix or gall bladder (“You do not absolutely need them.”), or replace a hip or knee (“You will be fine, after a while.”), or reconstruct an ulnar collateral ligament (“You will be better than ever.”), so we say “Sure. Sounds like a good idea.”, as though the patient was purchasing a vehicle or a sweater.

Here is a real example. For 30 years I suffered lower back pain (sciatica, radiculopathy). Each time I went through an incapacitating bout of this, I considered seeing a specialist. Because I was athletic and exercised regularly, I did physical therapy, and took pain relievers but nothing gave me permanent relief. Therefore, I spoke with (interviewed) orthopedists who were said to be experts but I never thought they were taking me very seriously or they spoke words that might have been in another language. At the beginning of last year, I mentioned all this to a friend who is an ophthalmologist and he said, “See Dr. X” [name omitted to protect the innocent]. I took his advice and had with me a copy of the lumbar magnetic resonance image I had had taken a few months earlier. When I visited his office, he explained the problem so that I could understand him clearly and he told me he could fix the problem in 20 minutes! This frightened me as much as had he told me he could not repair the problem, but he showed me the arthritic irregularities causing the spinal stenosis that was the problem. A week later, he smoothed the foramen through which the sciatic nerve passes and 20 minutes later he was done and I went home not long after. I did not need pain medication, not even an aspirin.

I am now, more than ever, convinced that all patients should be educated regarding their bodies, what strangers are plotting to do things to them, why it is important for patients to faithfully follow the instructions given to them, what to do and who to ask when s/he does not understand the instructions, and more. Of course, not all patients (perhaps not even most patients) can be or do not want to be educated but it might help if we tried. At the time of writing this, during the severe acute respiratory syndrome coronavirus-2 pandemic, we have multiple vaccines said to be effective against this virus and it will soon be obvious whether they are indeed effective. However, the public is even less confident about their effectiveness than are clinicians and scientific investigators, and questions regarding the efficacy of these vaccines seem to unduly, even negatively, influence their acceptance. How can we expect otherwise when some people are prescribed antibiotics for a given period and do not take them for the entire period suggested? Does anyone emphasize to them why they should take the complete dose, or are they simply told to “Do this”?

It seems to me that there is a need for more education. When I retired, I volunteered to teach reading to second graders (about 8 years old) for an hour twice a week in a school near my home. I know how to read and I thought it would be easy, and enjoyable for me, to teach the students. I was wrong. I began my first classes each year by telling them that I was not planning to teach them to read, that they already knew how to read (words), and that I would be teaching them what those words mean in the context of understanding the writer’s intent for the sentences.

They all did fabulously those years (although the real teachers and the school Principal asked me what “method” I was using and I told them that I was using an experimental method) and we (the students and I) all had fun. However, I realized that second graders knew just about nothing (not why one uses an adjective, not how you can tell when your vehicle is out of petrol, not what to do with money not spent, not why it matters when people are dying half a world away, not to lick a metal pole during the winter [or summer], etc.). Therefore, I educated them the best I could. At the end of each teaching year, they learned more than how to read words.

There is much for all of us to learn, simply to stay well-informed about our specialty, no matter what that is. Adding more to everyone’s schooling means we must make more than ordinary efforts. Unfortunately, we live in an age in which scientists no longer receive as much public respect as in the past, and some of our politicians manipulate the truth not only for their own benefit, but to the detriment of those they supposedly represent. For an obvious example, propaganda, lies, and pseudoscience were used by the previous US president when discussing the pandemic, which he said would disappear “like magic.” Many believed him and the pandemic inevitably resulted in a national and international disaster. Only education, including about Critical Thinking, Logic, and Reasoning, can overcome such nonsense. I suggest that those responsible for education consider adding these subjects to the current curriculum, which means adding the teaching of such subjects to the teachers’ syllabus at the school where they themselves are trained.

This likely would help improve the public’s understanding of and need for attention to details and, as important, to emphasize that with knowledge and background regarding what they are doing, and what people are doing to them, they will know why they are doing it. If nothing else, this could lead to lowered stress in patients. In addition, and of immediate value, we, the investigators and clinicians, might consider participating in frequent informal talks (not formal lectures) to public groups. These might include sessions on what it takes to become a laboratory scientist, what “an ordinary day in the life of a laboratory scientist or clinician” is like, how we recognize previously unknown viruses and other infectious agents and the diseases they cause, how vaccines are manufactured, why and how vaccines are first tested for safety and efficacy, which segments of the population might first be selected to receive new vaccines, how commercial manufacturers decide on pricing of their products (This might take a long night of bickering!), and so on.

My colleagues and I at Colorado State University and numerous others at other institutions throughout North America and elsewhere have been holding such sessions locally and state-wide and I believe we have been successful. Furthermore, educating the public, no matter the age group, makes our professional lives easier. Non-scientists are now asking informed questions, only some of which I can answer.

